# Role of Maternal Microbiota and Nutrition in Early-Life Neurodevelopmental Disorders

**DOI:** 10.3390/nu13103533

**Published:** 2021-10-09

**Authors:** Anissa Daliry, Evelyn Nunes Goulart da Silva Pereira

**Affiliations:** Laboratory of Cardiovascular Investigation, Oswaldo Cruz Institute, Oswaldo Cruz Foundation, Rio de Janeiro 21040-900, Brazil; evyspereira@gmail.com

**Keywords:** gut microbiota, neurodevelopmental disorders, obesity, gut–brain axis, maternal diet

## Abstract

The rise in the prevalence of obesity and other related metabolic diseases has been paralleled by an increase in the frequency of neurodevelopmental problems, which has raised the likelihood of a link between these two phenomena. In this scenario, maternal microbiota is a possible linking mechanistic pathway. According to the “Developmental Origins of Health and Disease” paradigm, environmental exposures (in utero and early life) can permanently alter the body’s structure, physiology, and metabolism, increasing illness risk and/or speeding up disease progression in offspring, adults, and even generations. Nutritional exposure during early developmental stages may induce susceptibility to the later development of human diseases via interactions in the microbiome, including alterations in brain function and behavior of offspring, as explained by the gut–brain axis theory. This review provides an overview of the implications of maternal nutrition on neurodevelopmental disorders and the establishment and maturation of gut microbiota in the offspring.

## 1. Introduction

Over the last 50 years, the prevalence of adult and childhood obesity has increased worldwide, reaching pandemic proportions [1]. Overweight and obesity affect over 1.9 billion adults, with 650 million of them being obese [2]. Obesity, which is the main risk factor for cardiovascular diseases, cancer, diabetes mellitus, and non-alcoholic fatty liver disease (NAFLD), accounts for more than 70% of early deaths worldwide and is the leading cause of mortality and premature disability [3].

The increase in the prevalence of obesity and other related metabolic diseases has been paralleled by an increase in neurological problems, both in adults and in children [4,5,6,7]. Epidemiologic studies have found a link between maternal metabolic diseases and offspring’s neurodevelopmental and psychiatric morbidity, including intellectual disability, cognitive impairment, autism spectrum disorders (ASD), and attention deficit hyperactivity disorder (ADHD). Obesity in mothers is linked to a 3.6-fold greater risk of intellectual disability or cognitive impairment in their children [8,9,10,11]. In addition, a positive association between high maternal body mass index (BMI) and ASD in offspring is widely observed (odds ratio range from 1.5 to 1.7) [12,13,14,15]. This risk is increased by preterm birth [14], high gestational weight gain [15], gestational or pre-gestational diabetes [12,13] and preeclampsia [16]. Large cohort studies have shown that ADHD symptoms in offspring increase in a dose-dependent manner accordingly to maternal pre-pregnancy BMI increase, from overweight to obese [17]. Other studies have reported a 1.6- to 2.8-fold greater risk of ADHD in offspring of obese mothers [18,19,20].

The exact etiology underlying neurodevelopmental disorders remains a challenge, but several genetic and nongenetic (exposome) factors are known to interact early in life to influence the risk for developing neurological diseases [21]. During pregnancy and early life, several environmental factors could influence the risk for developing neurodevelopmental disorders, including dietary pattern (healthy or unhealthy), socioeconomic aspects, infectious diseases, stress, pollutants, mode of delivery (vaginal or cesarean), and feeding pattern (breastfeeding or artificial feeding) [22]. In this scenario, a mechanistic knowledge of how the intrauterine environment of the mother and lactation may regulate offspring neurodevelopment is critical in exploring a causal relationship between these two phenomena. A potential mechanistic pathway linking maternal nutrition and offspring outcomes is the transmission of maternal microbiota. The human gut microbiota is made up of a complex and dynamic population of more than 10 trillion species, including bacteria, archaea, yeasts, and protozoa [23].

The gut microbiota acts as a metabolic organ, performing tasks not encoded in the human genome, such as producing metabolites not created by the human body [24] and delivering critical nutrients by degrading otherwise indigestible polysaccharides and sugars [25,26]. Furthermore, the gut microbiota has a role in the development, maturity, and maintenance of critical human systems such as the immune system [27], gastrointestinal tract function [26], and metabolism [28,29].

The concept of the gut–brain axis specifies a bidirectional signaling pathway between the gastrointestinal system and the central nervous system, linked by neurons of the sympathetic and parasympathetic nervous systems, as well as by circulating hormones and other neuromodulators [30]. Dysfunction of the gut–brain axis has been visualized as an essential mediator in the pathogenesis of neurodegenerative diseases [30,31,32].

As the microbiota is shared between the mother and child, maternal diet can influence the microbiota of the offspring. In the gastrointestinal system, a complex combination of mother and offspring factors generates a unique microbiome [33]. The concept of fetal microbiome, which postulates that microbial colonization begins in utero, remains controversial [34,35,36,37]; however, a wealth of evidence demonstrates that the microbiota of the mother can be seeded in the offspring at the time of birth via vertical transmission from the reproductive and gastrointestinal tracts [38,39,40,41,42] and can be transmitted by breastfeeding [43].

Therefore, promoting a balanced and diverse composition of the maternal gut microbiota is critical for establishing the first life bacterial community required to line the offspring on a healthy developmental trajectory. This review provides an overview of the complex network between diet-induced changes in intestinal microbiota and its possible links with neurodevelopmental disorders of the offspring. In this regard, we shed light on the dietary factors that affect the maternal and offspring microbiota and the pathways by which these changes affect the neurodevelopmental outcomes of the offspring.

## 2. Maternal Obesity and Child Neurodevelopmental Disorders

A recent meta-analysis showed that mothers who were overweight or obese before pregnancy had 17% and 51% higher chance, respectively, of having a child with poor neurodevelopmental outcomes. Children born to overweight/obese women had a higher incidence of ADHD (62%), cognitive or intellectual delay (58%), emotional or behavioral problems (42%), and ASD (36%) [44]. Ornoy and colleagues showed that school-age children born to mothers with diabetes or gestational diabetes exhibited neurobehavioral and motor disorders, mostly inattention and fine and gross motor impairment [45]. Studies have shown that the combined effect of maternal obesity and diabetes has more pronounced effects on the risk of ASD in children than the isolated effect of each condition [13,46].

The average score of children aged 6 to 9 years on the Bruininks–Oseretsky Test of Motor Proficiency correlated significantly with the level of maternal β-hydroxybutyrate (BHA) ketone bodies, suggesting prenatal and perinatal impacts on long-term neurodevelopmental course of diabetic mothers’ offspring [47].

Maternal elevated BMI is a risk factor for ADHD, but some authors suggest that it may not be a potential causal effect, as sibling comparisons showed significant attenuation of the previously observed associations, indicating that the association of maternal elevated BMI could at least, in part, be attributed to familial confounding factors [19,48].

Overweight and obese women have an increased risk of preterm birth (<37 weeks), which is a risk factor for neurodevelopmental disorders [49]. Preterm-born children evaluated at school age showed more than two-fold increased relative risk of developing ADHD and reduced cognitive test scores [50,51]. The mean cognitive scores at school age were directly proportional to the severity of prematurity at birth. The lower scores for motor, cognitive, and academic tests of preterm children at preschool age persisted in the primary school years [51]. Likewise, children of obese mothers with high birth weight (≥3.6 kg) had an increased risk of attention problems [52].

**Figure 1:**
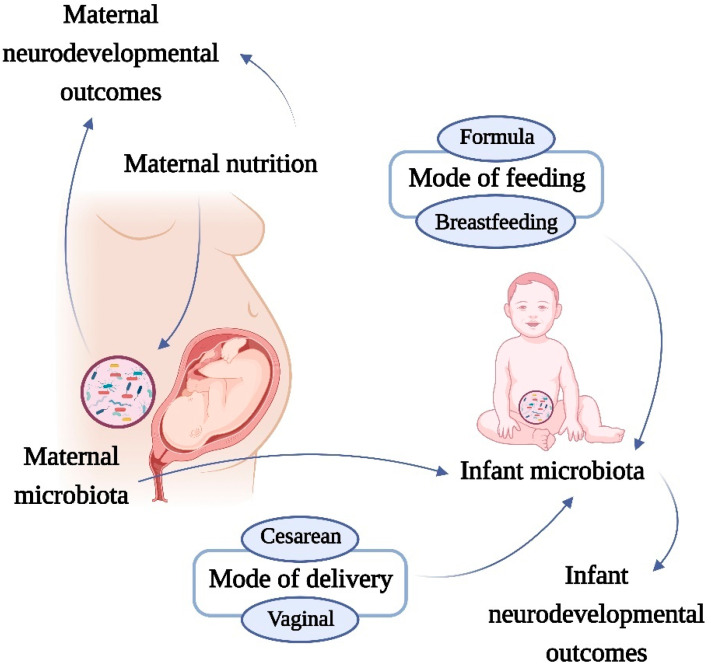
Effects of maternal microbiota and nutrition on neurodevelopmental disorders. The increase in prevalence of nutrition-related metabolic diseases has been paralleled by an increase in neurological problems, both in adults and in children. A possible mechanistic pathway linking nutrition and neurodevelopmental outcomes is the microbiota. Several factors can affect the neonatal microbiome. The microbiota is shared between mother and child, therefore the maternal diet, mode of feeding and mode of delivery can influence the microbiota of the offspring. This complex network involving early-life microbiota development can induce changes that link with the neurodevelopmental disorders of the infant.

Though the mechanisms that account for the development of brain dysfunction in the offspring of mothers with metabolic diseases are not fully understood, the metabolic capacities of the gut microbiota and the crosstalk with the developing brain during early life may play a pivotal role in this process (Figure 1).

## 3. The Effect of Maternal Diet on the Offspring Microbiota

**Figure 2:**
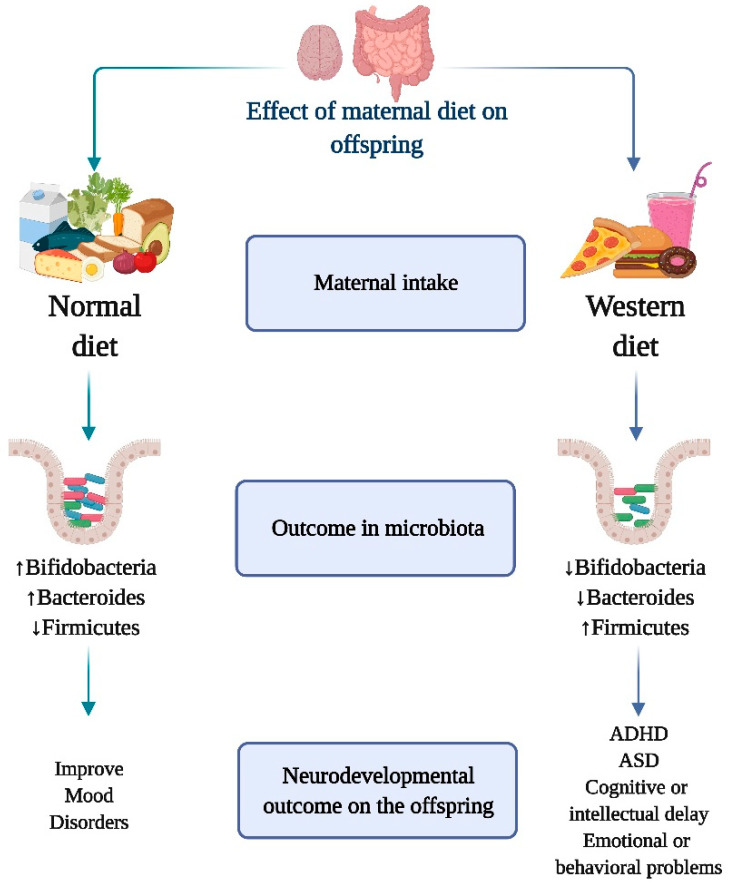
Role of maternal diet on the interaction of gut microbiota and neurodevelopmental outcome on the offspring. Increase in *Bifidobacteria* and *Bacterioids* and decrease in *Firmicutes*, is associated with the consumption of a healthy and nutritionally balanced maternal diet. This “healthy” dietary and microbiome profile has been linked to the prevention of neurodevelopmental problems and the onset of mental disorders. On the other hand, maternal intake of a diet rich in sugars, lipids and low in fruits and vegetables (Western diet) is associated with a decreased population of *Bifidobacteria* and *Bacterioids* increased *Firmicutes* species. The onset of neurodevelopmental illnesses such as attention deficit hyperactivity disorder (ADHD), autism spectrum disorder (ASD), cognitive or intellectual delay, and emotional or behavioral problems has been linked to the Western diet and its distinctive microbiome composition.

Microbiota modifications induced by recent changes in the human environment, such as the amount and composition of the diet, appear to be a key determinant of child health [53,54,55,56,57] (Figure 2). In fact, the link between food and health has been proposed since antiquity and can be exemplified by the concept of “let the food be thy medicine and medicine be thy food” postulated by the Greek physician Hippocrates, 2000 years ago. We now understand that nutrients can directly interact with microorganisms to boost or decrease their growth kinetics and to modulate their ability to extract energy from specific dietary elements, giving chosen members of the gut microbial community a direct competitive advantage [58] (Figure 2).

Importantly, changes in the mother’s diet prior to and during pregnancy can modify the infant gut microbiota [59,60,61]. According to the “Developmental Origins of Health and Disease” paradigm, environmental exposures (in utero and during early life) can permanently modify the body’s structure, physiology, and metabolism, resulting in higher risk and/or acceleration of disease in adulthood and across generations [62,63]. From an evolutionary perspective, maternal transfer of excess nutrients may have contributed to better offspring survival chances; however, nowadays, one of the most powerful predictors of childhood obesity is exposure to a scenario of gut dysbiosis of maternal origin, such as obesity and diabetes mellitus [64].

Cohort studies have demonstrated that imbalanced nutrition arising from excessive intake of nutrients during pregnancy increases the progeny’s vulnerability to metabolic disorders [65,66,67,68,69]. Microbial dysbiosis arising from a maternal high-fat diet was linked to a neuroinflammatory profile in the offspring’s hippocampus and amygdala, resulting in reduced social behavior and an anxiety-like phenotype in non-human primates and rodents [70]. Molecules that can cross the maternal compartment represent a risk to developmental programming in offspring, and an inadequate intake of macro-and micronutrients during pregnancy has been linked to an altered maternal microbiome [71] and poor neurocognition in offspring [72]. As a result, mother weight status, whether pre-pregnancy weight or excess gestational weight gain, may have major implications on neurodevelopmental outcomes in the offspring.

The Western diet is characterized by a high content of refined sugar, omega-6 polyunsaturated fats, and fast food, with an associated reduced consumption of fruits, vegetables, and fibers [73]. Several studies have explored maternal overweight- or obesity-associated changes in the microbiome of the early-life gut, and the consumption of high-fat diets is a common cause of macronutrient consumption imbalance during pregnancy (Figure 2). The maternal and offspring microbiota in humans and rodents are influenced by the type of diet consumed prior to or during pregnancy [74,75,76]. Fecal *Bacteroides* and *Staphylococcus* concentrations increase during the first 6 months of life in infants born to overweight mothers, while bifidobacterial concentrations are higher in infants from lean mothers [76]. Studies in Yucatan pigs revealed that a maternal Western diet during pregnancy and lactation, even in the absence of obesity, can modify the microbiota of the offspring and have significant consequences on blood lipid levels, gut–brain axis, and neurocognitive skills after weaning [77]. Maternal exposure to a Western diet generated alterations in the intestinal microbiota composition of the offspring in suckling mouse pups, including a higher Firmicutes/Bacteroidetes ratio and a significant decrease in relative abundance of *Akkermansia*, a Verrucomicrobiaceae member, which lives in the outer mucus layer of the colon and has been proposed as “healthy” intestine marker [74].

Mechanistically, a high-fat diet prior to and during pregnancy has been shown to impair maternal hypothalamic–pituitary–adrenal axis plasticity and hypothalamic gene response to stress in the offspring [78,79]. The offspring of mothers exposed to a Western diet showed significant changes in the expression of crucial genes responsible for normal functioning of the intestine, including cholecystokinin hormones, ATP-binding cassette transporter A1, fructose transporter GLUT5, and immune response/inflammation-related genes such as Ly86, Cxcr6, and CD74, indicating that mothers’ food choices are critical for the proper development of the intestine in their offspring [74]. Together, these findings support the importance of counseling pregnant mothers about their nutritional needs during pregnancy and lactation, and emphasize the importance of diet-induced gut microbiota modulation (Figure 2).

## 4. Early-Life Microbiota Colonization

### 4.1. Birth Mode

Cesarean section rates have exponentially increased around the world in recent years and at present, it exceeds the World Health Organization (WHO) recommendations [80]. This may lead to several implications, as the mode of delivery may affect the early life microbiota, leaving a lasting imprint on the biology of offspring [81]. The mother cervicovaginal and fecal microbes dominate the microbial inoculum of vaginally delivered neonates, whereas cesarean birth imparts unique microbial consortia and are frequently dominated by skin microorganisms [40,41,42].

Many studies have revealed correlations between birth mode and neurodevelopmental disorders in the offspring. Cesarean delivery has been linked to an increased risk of neurodevelopmental and psychiatric outcomes, including ASD (33% increased odds) and ADHD (17% increased odds), intellectual disabilities, obsessive compulsive disorder, tic disorders, and eating disorders [82,83]. However, causality has not yet been confirmed, as confounding factors should be taken into account, including familial factors [84,85,86,87,88], and that emergency C-Section is often the result of pregnancy (e.g., preeclampsia and gestational diabetes) and/or birth (e.g., fetal distress, dystocia, and failed induction) complications, which could also affect brain development. In addition, there is a potential genetic overlap between psychiatric disorders and the likelihood of delivery by C-section [89,90].

In the first months of life, *bifidobacteria* are more abundant in children exposed to microbes via vaginal delivery than in children born via cesarean delivery, who have more *Enterococcus* spp. and *Klebsiella* spp. [81,91,92]. The modulation of host defensive responses is a unique benefit of *bifidobacteria*; acetate produced in large amounts by preventative *bifidobacteria* exerts action on the colonic epithelium by generating anti-inflammatory and anti-apoptotic effects [93]. In contrast, cesarean delivery is associated with a higher population of potentially pathogenic microorganisms, which are commonly associated with the skin, oral cavity, and hospital environment [40,94], and increased levels of pro-inflammatory markers, which are linked to the offspring’s development of depression, learning, and memory deficits [82,95,96].

Reduced levels of intestinal *Bacteroides* spp., associated with C-section delivery, have significant influence on human physiology and metabolism. Alterations in gut microbiota of offspring by synergic birth factors also increases the risk of developing metabolic diseases such as obesity and type 1 diabetes [97,98]. A preclinical study demonstrated that mice delivered by C-section exhibited social, cognitive, and anxiety problems in early life, and the *Bifidobacterium breve* strain supplementation was able to reverse these selective behavioral alterations [99]. A cross-sectional study of 8900 preschool children found that the odds of being overweight and obese were 1.35 and 1.25, respectively, in children delivered by C-section [100]. In a cohort study of 935 overweight and obese mothers, both vaginally and cesarean-delivered offspring had three and five times more risk, respectively, to become overweight at ages 1 and 3 years, respectively, compared with offspring born vaginally to a mother of normal weight. Although several other factors may influence childhood obesity, such as genetic components [101] and psychological and psychiatric components that may affect maternal and child feeding habits [102,103], it is intriguing that the likelihood of being obese, in children born to obese mother, is higher in children delivered by C-section than in children delivered vaginally, suggesting that the risk of childhood obesity may depend on the mode of delivery. A higher Firmicutes species richness was observed in the microbiota of children of overweight mothers; however, the participating genera of *Lachnospiraceae* differed between vaginal and cesarean deliveries. Therefore, the birth mode and infant gut microbiota in the offspring gut may act as sequential mediators of the association between maternal pre-pregnancy and pregnancy metabolic status and child overweight and/or neurodevelopmental disorders [42].

These findings suggest that birth mode alters intestinal microbiota, leading to various metabolic consequences, including obesity and metabolic dysfunction. Furthermore, C-section may disturb the maturation of the microbiota–brain–gut axis, altering developmental trajectories and perhaps leading to the emergence of neurodevelopmental and other brain diseases later in life [32,104,105,106,107,108] (Figure 1).

### 4.2. Infant Feeding Mode

Breastfeeding is recommended by the World Health Organization (WHO) and the American Academy of Pediatrics (AAP) as the best source of nourishment for newborns throughout the first six to twelve months of life [109,110]. Breastfeeding has been proven in several studies to provide short- and long-term benefits, including protection against cognitive development, ADHD, and ASD [111]. Breastfeeding has also been studied for its emotional benefits, which have been linked to increased oxytocin levels [112] and improved mother-infant bonding [113,114], both of which promote greater maternal sensitivity and responsiveness [115], which has independent effects on maternal caregiving and infant brain development.

Feeding type (whether breastfed or formula fed) is another major factor that affects the development and complexity of the microbiota [116] (Figure 1). Studies have shown that human milk is not sterile and that maternal microbiota and other factors in breastmilk are a continuous source of colonizing microbes to the offspring’s gut, modulating microbiota composition and immunological function in offspring, and can influence neural development [117].

Breastfeeding is the dietary element that is most consistently associated with gut microbial diversity in offspring when compared to formula feeding [118]. Formula-fed newborns, on the other hand, show a decline in bacterial variety and richness even after the first year of life [41]. The presence of the same bacterial strain in breast milk and the feces of breastfed infants indicated the mother-to-child microbiota transmission [119]. Martín et al., in a study with 10 mother–infant pairs, showed that the genera that were found in both infant feces and human milk samples accounted for 70–88% of the overall relative abundance in infant feces samples, confirming the notion of vertical bacterial transmission from milk to the infant gut. In addition, *Bifidobacterium breve* and *Lactobacillus plantarum* bacteria with identical characteristics were recovered from milk and infant feces samples [120].

Viable skin and non-skin Gram-positive bacteria are present in the breast milk. Streptococci of the mitis and salivarius groups, as well as coagulase negative staphylococci, predominate both in human milk and feces of breastfed newborns [92,121,122,123,124]. These microorganisms, which are less common in formula-fed newborns, may be able to prevent the establishment of undesired pathogens in the infant gut [125,126,127]. Bacteroides and Bifidobacterium have a higher relative abundance in the gut bacterial population of exclusively breastfed infants [128]. A large longitudinal study involving 903 children aged 3 to 46 months showed that breastfeeding was linked to greater levels of Bifidobacterium species (*B. breve* and *B. bifidum*). The discontinuation of breastfeeding led to alteration of the microbiome, resulting in a rapid maturation of the gut microbiome, as indicated by the increase in species of the phylum Firmicutes [129].

Human milk is also rich in nutrients, among which oligosaccharides are the third most abundant after lactose and lipids. Human milk oligosaccharides can act as prebiotics, triggering the growth of particular bacterial groups, such as Bifidobacterium, with more than a two-fold increase in the number of bacteria compared to the microbiota of formula-fed infants [130].

Maternal obesity may influence offspring microbiota and neurodevelopmental disorders through different mechanisms. Maternal obesity can result in the prevalence of pro-inflammatory fatty acids in breast milk beyond those levels critical for offspring neurodevelopment. Compared to breastmilk from lean mothers, breastmilk from obese mothers had a higher omega-6 to omega-3 fatty acid ratio and lower concentrations of eicosapentaenoic, docosahexaenoic and docasapentaenoic acids, and lutein, which together induces a pro-inflammatory state and decreases neuroprotective properties [131]. Higher concentrations of fatty acids, such as docosahexaenoic acid, have been linked to improved visual and language development [132].

Considering the breastfeeding–gut–brain axis mechanism, both breast milk microorganisms and prebiotics play critical role in the development of the microbiome in the offspring, leading to neurodevelopment- associated outcomes. In a preclinical study, Tarr et al. demonstrated that milk oligosaccharides sustain normal microbial population and behavioral responses following stressor exposure, possibly through effects on the gut microbiota-brain axis, and maintain normal behavior on tests of anxiety-like behavior and normal numbers of doublecortin-positive immature neurons [133].

Although the mechanisms behind breastfeeding-induced beneficial effects are not fully explained, it is possible that the standard microbial environment provided by breast milk versus formula feeding may play a key role in offspring neural development. Therefore, the mother’s commitment to a healthy and balanced diet, associated with breastfeeding, will have significant positive impacts on infant microbiome, and prevent the onset of neurodevelopmental problems.

## 5. Microbiota as a Metabolic Factory

The microbiota can be viewed as an active ‘‘organ’’ with metabolic capacities that we did not have to develop on our own. These functions include the ability to handle indigestible food components such as plant polysaccharides, synthesize vitamin K and other vitamins belonging to the B complex, supplying essential components [134,135]. Gut microbes metabolize polysaccharides and complex carbohydrates to short-chain fatty acids (SCFAs), such as butyrate, acetate, and propionate [136], which can be used as energy sources by other organs and cells. Colonocytes use butyrate as their principal energy source, while propionate and acetate are utilized by the liver for lipogenesis and gluconeogenesis [137,138].

Seminal studies have shown that germ-free (GF) animals have lower total fat content than conventionalized or fecal-transplanted animals [134], suggesting that microbiota can affect host fat storage and energy metabolism [134]. Microbiotas participate in host fat storage by increasing dietary polysaccharide processing, and elevating key enzymes and proteins involved in de novo hepatic lipogenesis, such as acetyl-CoA carboxylase (Acc1), fatty acid synthase (Fas), sterol response element binding protein 1 (SREBP-1), and carbohydrate response element binding protein (ChREBP). As a result, higher triglyceride content in the liver and increased fat storage in adipocytes promote the storage of calories obtained from the diet into fat [134,139].

While more than 90% of bacteria comprising the mouse and human distal gut microbiota are members of the Bacteroidetes and Firmicutes phyla [140,141,142,143], obesity is linked to a higher relative abundance of Firmicutes and a lower relative abundance of Bacteroidetes [140,141]. Furthermore, when a lean diet is substituted, obese people lose weight and restore Bacteroidetes [69,140,144]. Studies on *ob/ob* mice, diet-induced obese mice, and humans have demonstrated the presence of an obesity-associated gut microbiota with higher ability for energy harvesting from the food [69,144]. Western diet-fed mice were enriched for the Mollicute lineage *Eubacterium dolichum* within the Firmicutes phylum, which reached an average of 70% of the gut microbiome [144]. The colonization of GF mice with microbes from an obese donor, resulted in significantly greater adiposity, providing the first evidence that differences in the murine gut microbiome are sufficient to alter host body composition [69].

Questions remain to be answered on the mechanisms that mediate the association between the relative abundance of Bacteroidetes to Firmicutes divisions and obesity, and to what extent this relationship can self-perpetuate. Changes in the population dynamics, interactions, and distributions of microbiota caused by obesity may act as an ‘‘environmental’’ factor that leads to a predisposition toward higher energy harvest from the diet and increased storage in tissues. This obesity-related microbiome may contribute to increased infant adiposity, which may lead to elevated inflammatory state and neurotoxicity, and thus contribute to a compromised neurodevelopment [145,146,147,148,149,150,151,152].

## 6. Important Bioactive Nutrients and Their Association with the Gut Microbiota

### 6.1. Micronutrients

Deficiencies in micronutrients are a global health challenge, and the gut microbiome is malleable and varies significantly due to micronutrient composition. Micronutrients are critically important for cellular development and differentiation, metabolism, immune system function, and neurological development during fetal, newborn and infant growth.

Preclinical studies with GF animals demonstrated that the gut microbiota plays a role in iron uptake and storage [153,154]. Hibberd MC et al. [155] showed that vitamin A deficiency has a huge effect on gut microbial community, causing an abundance increase in *Bacteroides vulgatus*. Studies in rodents and humans have demonstrated impairments in neurocognitive development due to deficiencies in micronutrients including zinc [156,157,158,159,160], iron [161,162,163,164], and B vitamins [165,166,167,168]. A study analyzing the oral, gut, vaginal, and breast milk microbiotas in pregnant women provided with a daily micronutrient-supplemented probiotic yogurt reported an increase in the relative abundance of *Bifidobacterium* and a decrease in *Enterobacteriaceae* in the feces of newborn [169]. Glycans in milk promote bifidobacterial growth, and specific milk oligosaccharides increase following micronutrient/probiotic supplemented induced by yogurt consumption [169]. Folate, the natural form of vitamin B9, is an essential cofactor in a variety of biological pathways, including nucleotide biosynthesis and DNA methylation [170]. Folate deficiency during pregnancy has been associated with increased neural tube defects, such as spina bifida, in offspring [171]. Besides dietary sources, intestinal bacteria in the colon can produce substantial amounts of folate as well as other B-vitamins [172]. In fact, more than 18% of the folate requirement can be fulfilled by bacterial folate biosynthesis [173]. The identification of a proton-coupled, high-affinity folate transporter in the human colon implies that bacterially biosynthesized folate can be absorbed and participate in host metabolism [174]. Folate deficiency has been associated with sensory axonal neuropathy [175,176] and psychiatric features, including cognitive decline, cognitive deficits, depression, and schizophrenia [177,178,179]. In rats with Alzheimer’s disease-like dementia, folate and vitamin B12 deficiencies additively impaired memory function by blunting hippocampal insulin signaling and modifying the gut microbiota [180].

Magnesium (Mg) modulates neuronal transmission and neuromuscular coordination in the brain, is a cofactor for a variety of enzymes, as well as participates in RNA, DNA, and protein stability [181,182]. Mg protects against excitotoxicity and neuronal cell death by blocking the calcium channel in the *N*-methyl-d-aspartate (NMDA) receptor [181,183,184,185]. Brain Mg concentration enhances synaptic plasticity which affects learning and memory skills [186,187]. Magnesium (Mg) deficiency is linked to an inflammatory and oxidative state characterized by increased in IL-6 and TNF-α, while Mg supplementation induces changes in microbiota that are associated with protective effects [188]. The content of *bifidobacteria*, and to a lesser extent of lactobacilli, decreases in the cecum during short-term Mg deficiency, independent of any significant change in other nutrient intake [188]. In a clinical study, Effatpanah et al. showed that subjects with ADHD had lower serum Mg levels compared with healthy controls [189].

Zinc is a structural component of many proteins and a cofactor of over 300 enzymes that regulate a variety of cellular functions and cellular signaling pathways important for brain and body health [190]. Zinc is also found in synaptic vesicles in the brain, especially in glutamatergic terminals [191,192,193]. Moreover, Zinc is involved in neuronal activity and affects the activity of N-methyl-D-aspartate (NMDA), α-amino-3-hydroxyl-5-methyl-4-isoxazole-propionate (AMPA), GABAA, glycine inotropic [194] and GPR39 receptors [195]. In physiological concentrations, Zinc has neuroprotective properties but is neurotoxic in excessive amounts [196,197,198,199]. As a result, an imbalance in zinc homeostasis has harmful effects on a range of brain processes, eventually leading to the neurodevelopmental disorders and psychiatric diseases [200]. Evidence from animal models has shown that the gut microbiome is able to collect zinc from the host cells when this element is scarce, which in turn influences the host-microbiota composition, leading to inflammation of the intestinal wall [201]. Zinc deficiency in pregnant mice has been associated with abnormal gut–brain signaling by altering gut physiology, being linked to microbiota dysbiosis and triggering an increase in inflammatory markers such as IL-6, IL-1β, and CCL2 [202]. Zinc supplementation combined with treatment effectively improved the symptoms of the attention-deficit disorder subtype of ADHD in 150 children aged 6 to 15 years in a randomized, double-blind clinical trial [203].

The vitamin D receptor (VDR), vitamin D metabolites, and enzymes important for vitamin D activation have been detected in the brain and central nervous system [204] suggesting that vitamin D is associated with cognitive function. Experiments have also shown that active vitamin D influences brain and neuron development [205] and has neuroprotective and antioxidant properties [204]. In addition, vitamin D deficiency has been associated with ageing, behavioral, social, motor, and sensory deficits [206,207,208,209]. Moreover, in humans, vitamin D has been shown to modulate host immune responses against certain bacteria and affect the composition of the gut microbiota. Multiple sclerosis, autism, and Alzheimer’s disease, among other neuroinflammatory illnesses, characterized by potential microbiome abnormalities, have been linked to vitamin D deficiency [210,211,212]. In a meta-analysis, Shen and Ji reported that subjects deficient in vitamin D were at an increased risk of developing Alzheimer’s disease [210]. Mostafa and Al-Ayadhi determined that children with autism had significantly lower vitamin D serum levels than healthy children [213]. Evidence supports the critical role of vitamin D in the maintenance of gastrointestinal homeostasis, microbiota composition, and regulation of mucosal inflammatory responses [214]. Vitamin D also has proven effects on the modulation of pattern recognition receptors [215] and maintenance of the intestinal barrier function [216,217]. Increased serum vitamin D was associated with a decreased Firmicutes to Bacteroidetes ratio, increasing beneficial bacteria, and decreasing pathogenic bacteria [218]. Mechanistically, vitamin D deficiency is associated with increased expression of Paneth cell defensins, tight junction genes and mucin-2, and decreased levels of circulatory lipopolysaccharide [219]. In addition, the gut microbiota and vitamin D deficiency in prenatal and early life can act synergistically to affect the development of obesity [219].

### 6.2. Polyunsaturated Fatty Acids

Omega-6:omega-3 (n-6:n-3) long-chain polyunsaturated fatty acids (LC-PUFAs) play an important role in the brain’s physiological activities. While n-3 regulates neurotransmitter production, transport, and release, n-6 is involved in signal transduction [220,221,222]. When n-6 intake exceeds n-3 LC-PUFA intake, the former replaces the latter in the neuron membrane, changing function and causing inflammation [223]. Western diet consumption is linked to a greater n-6:n-3 ratio and the increased incidence and prevalence of overweightness and obesity [224]. A high prenatal and perinatal n-6:n-3 LC-PUFA ratio is associated with worsening of ADHD symptoms [225,226] and slower psychomotor and mental development [227,228] and may impair language, cognitive, psychomotor, and social development [229,230] at infant and child stages. Even though lower proportions of n-3 LC-PUFAs in children with neurodevelopmental disorders have been reported, the molecular mechanisms that link LC-PUFA status and behavioral problems are still not fully understood. As gut microbes metabolize PUFAs derived from dietary fat [231,232,233,234,235], which function as a detoxifying mechanism in the gastrointestinal tract [236], and exert anti-inflammatory effects [237,238,239,240]; it may be postulated that gut microbial PUFAs are important metabolites in the prenatal and perinatal periods to prevent behavioral problems. Further studies are necessary to elucidate the effects of maternal LC-PUFA status during pre-pregnancy and pregnancy on the maternal and offspring microbiota and their association with neurodevelopmental disorders in childhood.

### 6.3. Prebiotics and Probiotics

According to the Food and Agriculture Organization of the United Nations (FAO) and WHO, probiotics are strictly selected microorganisms which, when adequately administered, provide important improvements to the host’s health. Prebiotics, on the other hand, are non-viable food components that provide a health benefit to the host through modulating the microbiota, an alternative to probiotics or as an additional support, improving the survival of probiotic microorganisms, particularly *Lactobacilli* and *Bifidobacterium*, in the gastrointestinal tract.

The close relationships between the maternal–fetal gut microbiota axis and health/disease have aroused great interest in exploring probiotics and prebiotics to favorably influence and establish gut microbiota homeostasis [241].

GF mice showed decreased anxiety-like behavior and increased motor activity in studies [242,243,244] indicating the modulation of conventional gut microbiota has an impact on behavior development, as well as neurochemical changes in the brain. It is possible to improve anxiety and depression using probiotics-based therapy. Oral administration with the human commensal *Bacteroides fragilis* was able to improve communicative, stereotypic, anxiety-like, and sensorimotor behaviors of the offspring of the maternal immune activation mouse (MIA) model, as well as modify gut permeability and microbial composition. The prenatal administration of oral probiotics protected the offspring from developing ASD-like behaviors induced by maternal immune activation, preventing (i) the increase in the IL-6 and IL-17a levels in both maternal serum and fetal brains, (ii) loss of parvalbumin positive neurons, and (iii) the decrease in γ-aminobutyric acid levels in adult´s offspring prefrontal cortex [245]. Maternal probiotic treatment with *Lactobacillus acidophilus* and *Bifidobacterium infantis* modified the neonate’s microbiota in C57BL/6J mice, improving the neurodevelopmental outcomes of offspring [246]. At pre-weaned age, maternal supplementation reduced postnatal peripheral inflammation, restored impaired blood–brain barrier permeability, and normalized tight junction protein expression. Furthermore, maternal probiotic supplementation influenced leukocyte trans-endothelial migration, extracellular matrix damage, and neuroinflammation. Reduced astrocyte/microglia activation and downregulation of the transcriptional regulators CCAAT/enhancer binding protein delta (CEBPD) and nuclear factor of kappa light polypeptide gene enhancer in B-cells inhibitor, alpha (IκBα), were linked to improved neurodevelopmental outcomes in offspring, probably due to promoted neuronal and oligodendrocyte progenitor cell development [246].

The prebiotic inulin reduced reactive oxygen species levels, protein and lipid peroxidation, and cholinesterase activity in the cerebellum, cortex, and striatum of maternal and fetal brains in a developmental model of rotenone-induced neurotoxicity, suggesting a potential role for indigestible oligosaccharides in reducing oxidative stress-mediated developmental origins of neurodegenerative disease.

In a developmental model of rotenone-induced neurotoxicity, prebiotic inulin reduced reactive oxygen species, protein and lipid peroxidation, and cholinesterase activity in the cortex, cerebellum, and striatum of maternal and fetal brain, suggesting a possible role for indigestible oligosaccharides in reducing oxidative stress-mediated developmental causes of neurodegenerative disorders [247]. Schmidt et al. investigated the effects of the prebiotic Bimuno^®^ galactooligosaccharide on the release of cortisol, stress hormones, and emotional processing in healthy subjects. Ingestion of Bimuno^®^ galactooligosaccharide decreased salivary cortisol awakening response and reduced attentional vigilance to negative versus positive information. The changes in mood may be related to the bifidogenic effects of galactooligosaccharide supplementation [248].

Thus, data collectively support the influence of probiotic and prebiotic intake on gut–brain axis communication via intestinal microbiota alterations, affecting brain function and behavior.

## 7. Conclusions

One promising link between maternal microbiota and offspring neurodevelopmental outcomes is maternal nutrition, as the intrauterine and early life phase are critical early windows in which the colonization of the gut microbiota occurs, which can significantly affect infant health outcomes. Clinical and preclinical studies suggest that maternal nutrition can interact with the natural trajectory of the offspring microbiota and play a role in brain health programming. The neurological system and the intestinal flora have concurrent developmental trajectories, and at this moment, maternal diet patterns can act, leaving a distinctive fingerprint in the infant intestinal microbiota and affecting offspring´s brain function and behavior. Many of the studies included in this review, however, were epidemiological studies, which have issues with validity, accuracy, and interpretation, and frequently fail to show a causal relationship between the phenomena studied. Therefore, further studies that categorically establish a causal relationship between maternal nutrition, offspring microbiota modulation, and its consequences in early life neurodevelopmental disorders are necessary. Understanding the role of maternal diet on microbiota modulation and its consequences on neurodevelopmental outcomes in early life can open new perspectives and potential targets for modulatory interventions of the offspring microbiome, aiming to reduce the risk of neurodevelopmental disease.
